# Supplementary material for: Role of PRMT5 in regulating ferritinophagy during Mycobacterium tuberculosis infection
Source: PLoS Pathog. 2026 Sep 22;22(9):e1014614. doi: 10.1371/journal.ppat.1014614 (PMC13630314; doi:10.1371/journal.ppat.1014614)
Supplement: Supplementary file 1 [file ppat.1014614.s009.pdf]

FTH1

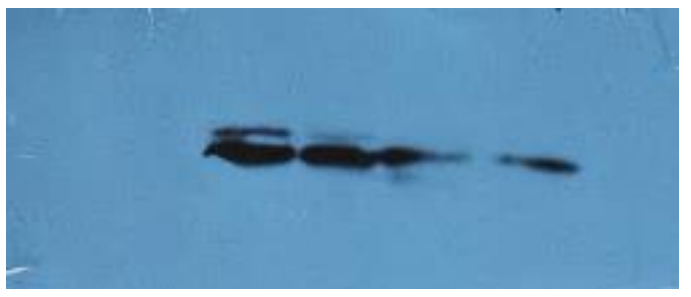

ACTB

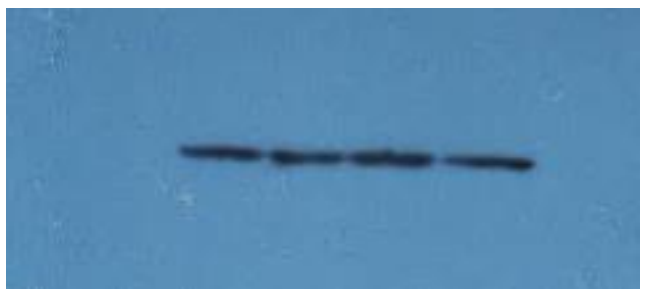

Uncropped blots used in Figure 2 Panel B

FTH1

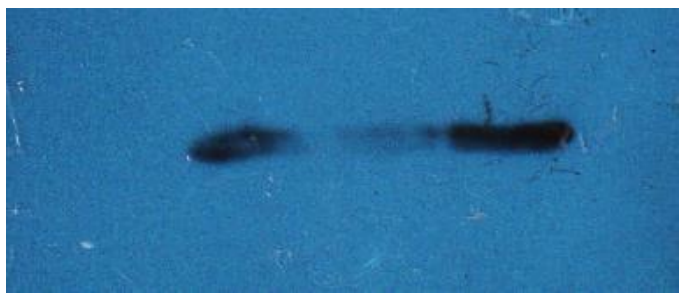

ACTB

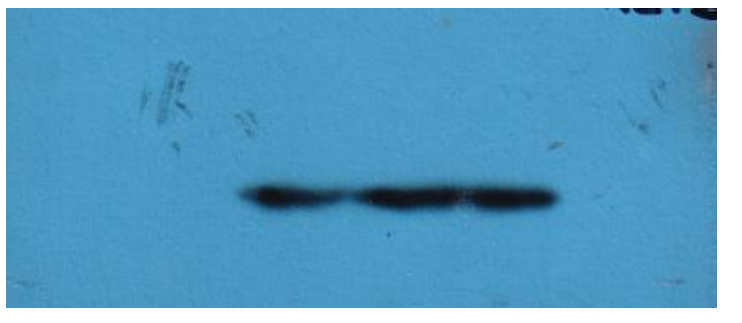

Uncropped blots used in Figure 2 Panel C (left)

FTH1

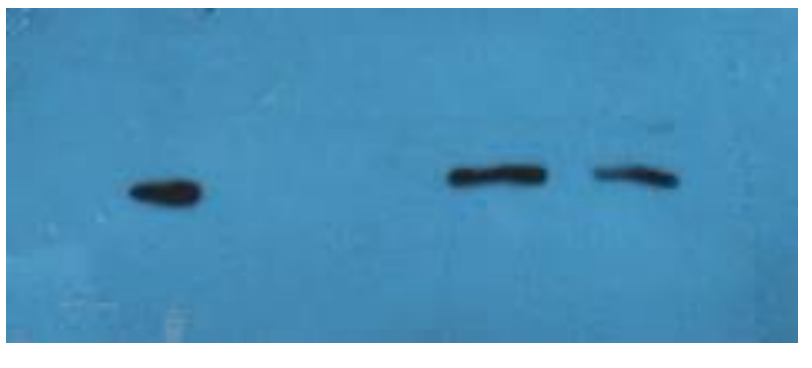

ACTB

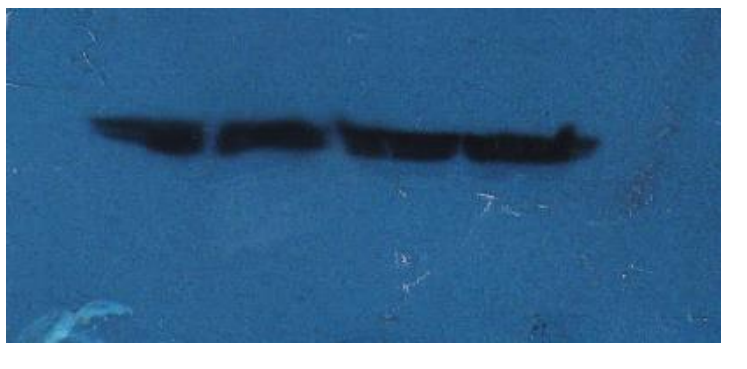

Uncropped blots used in Figure 2 Panel C (right)

FTH1

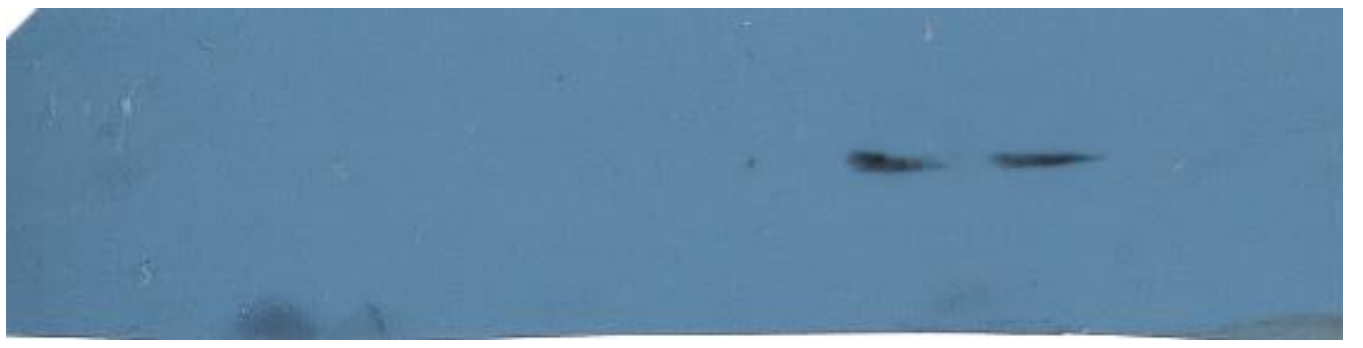

NCOA4

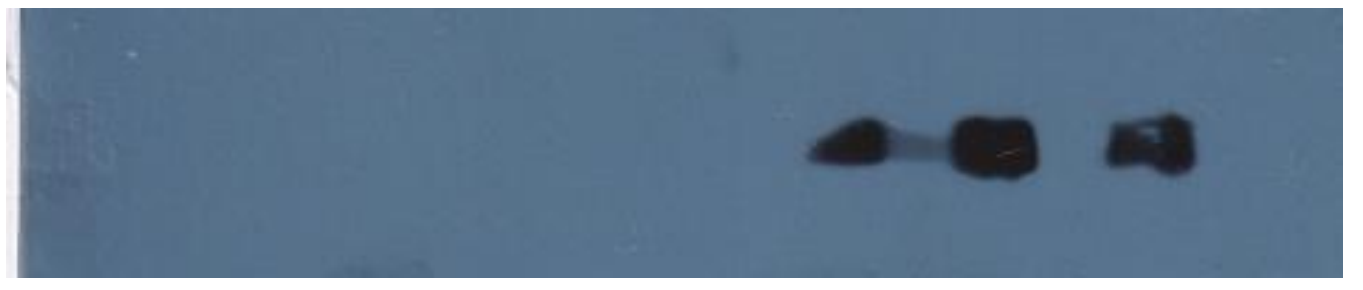

INPUT

FTH1

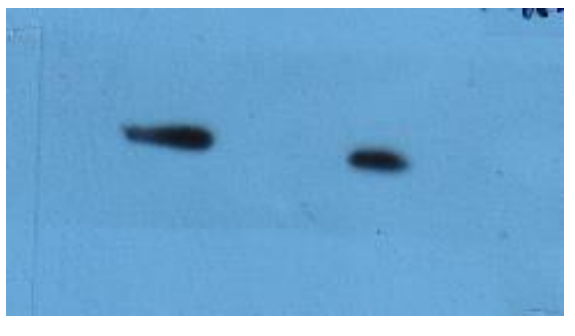

NCOA4

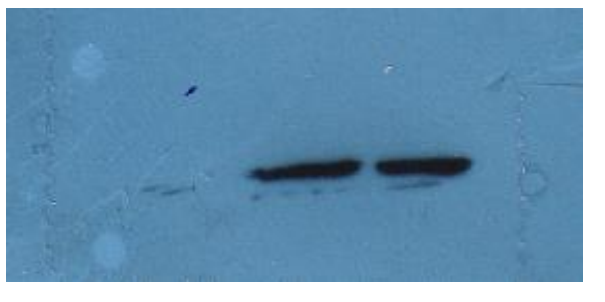

ACTB

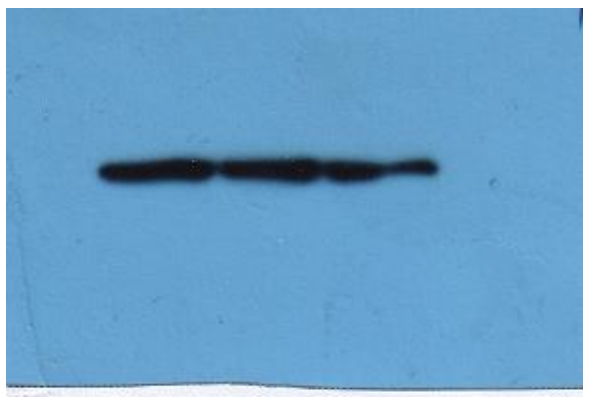

Uncropped blots used in Figure 3 Panel A

NCOA4

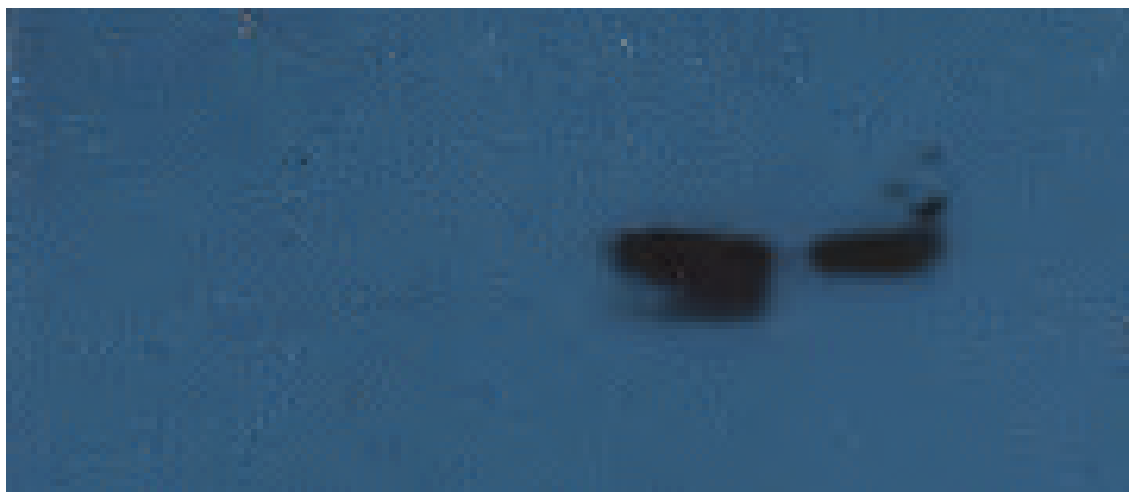

PRMT5

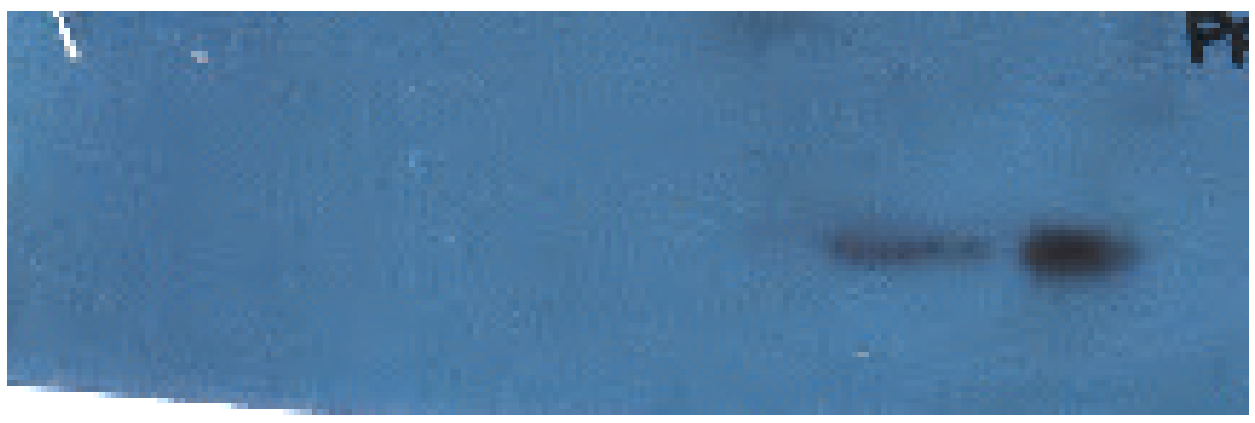

SDMe-R

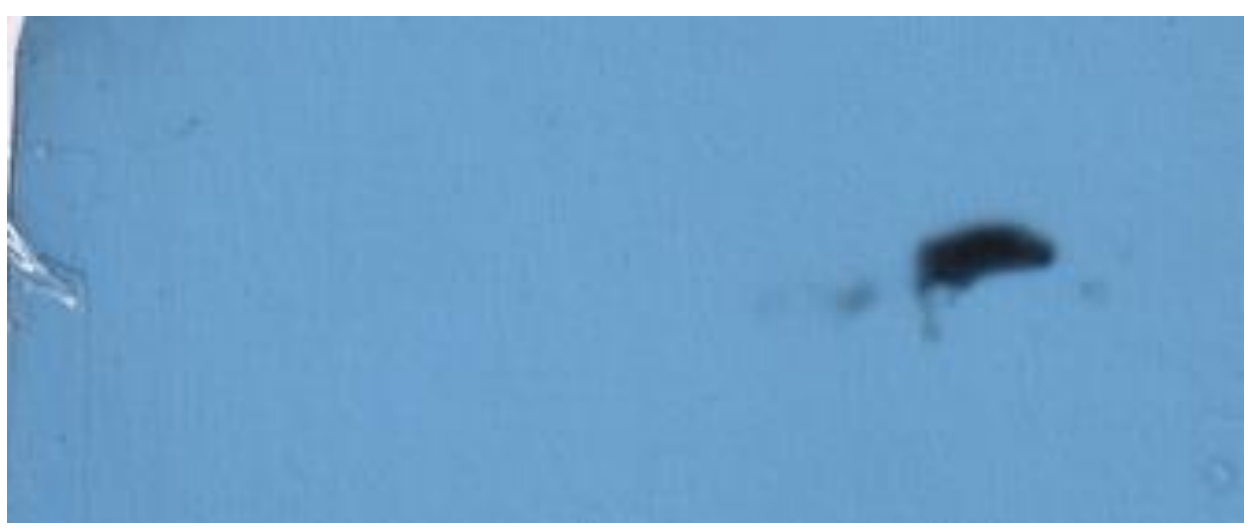

INPUT

ACTB

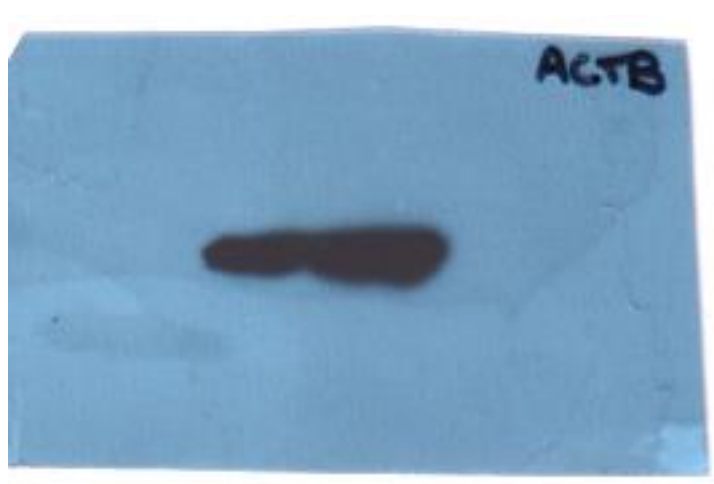

NCOA4

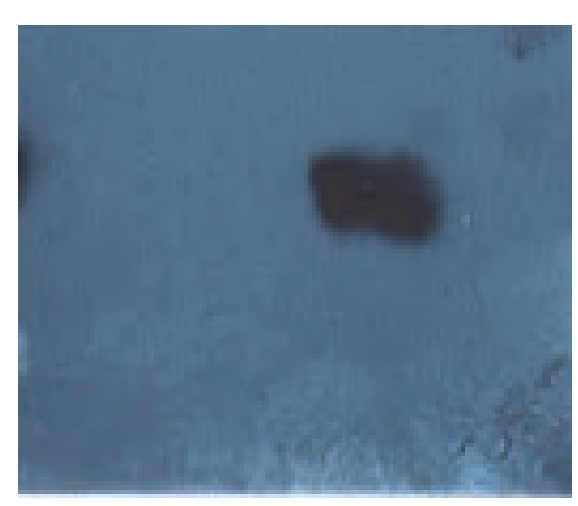

Uncropped blots used in Figure 3 Panel C

NCOA4

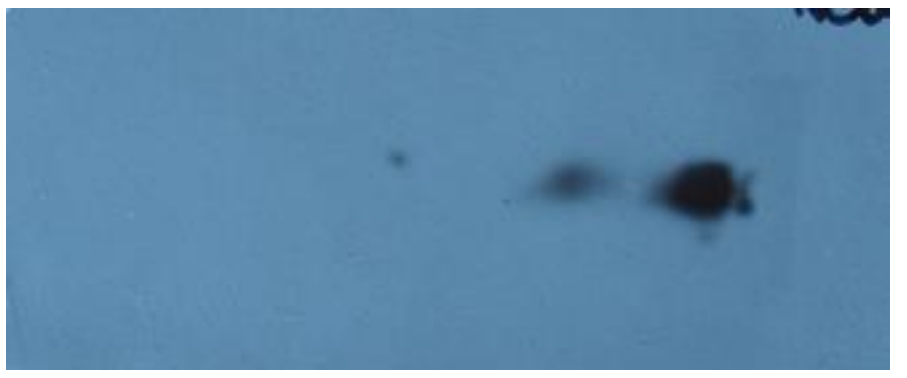

PRMT5

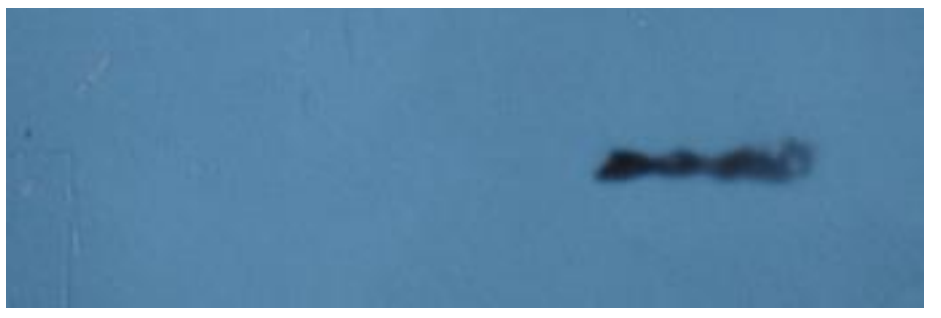

INPUT

ACTB

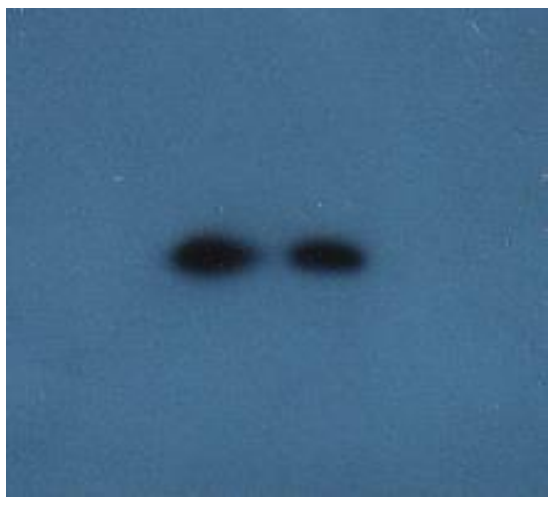

PRMT5

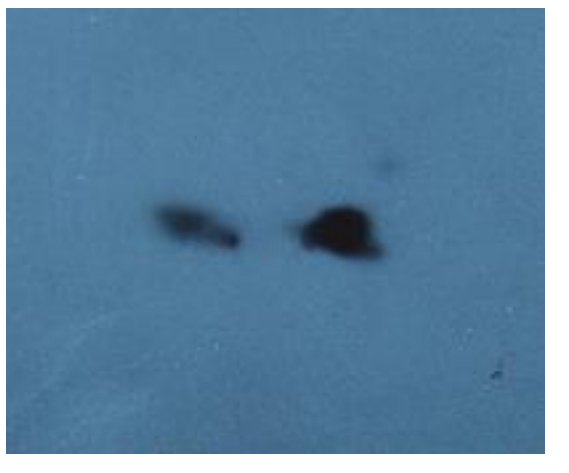

Uncropped blots used in Figure 3 Panel D

NCOA4

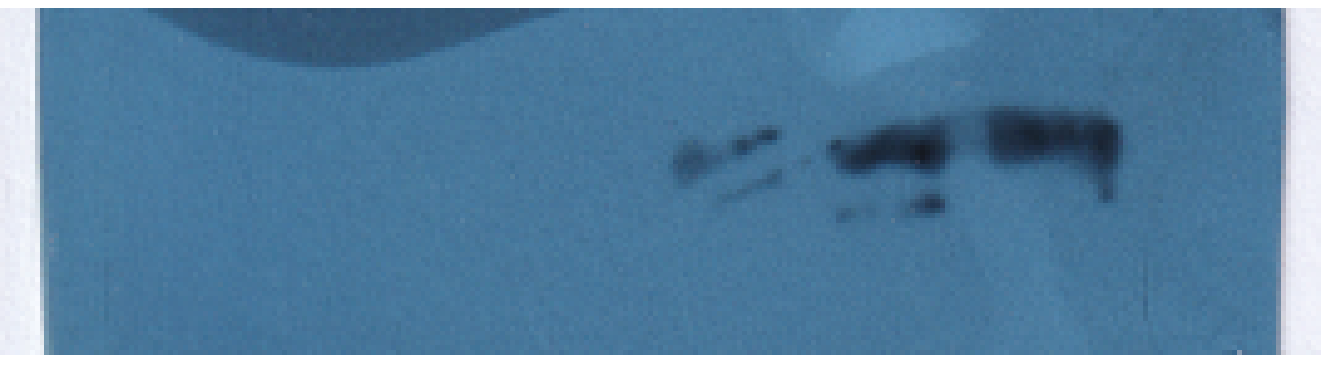

SDMe-R

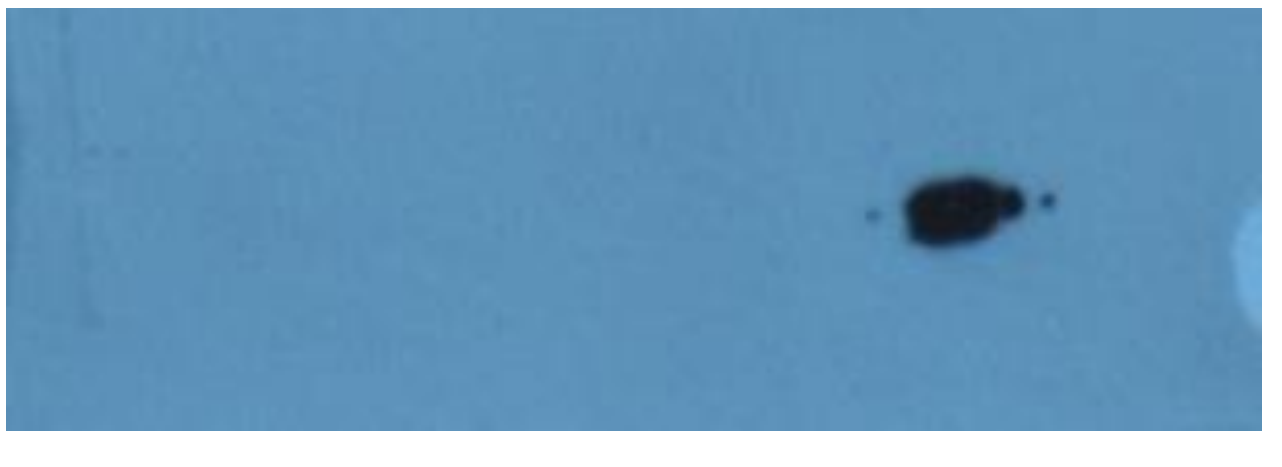

INPUT

ACTB

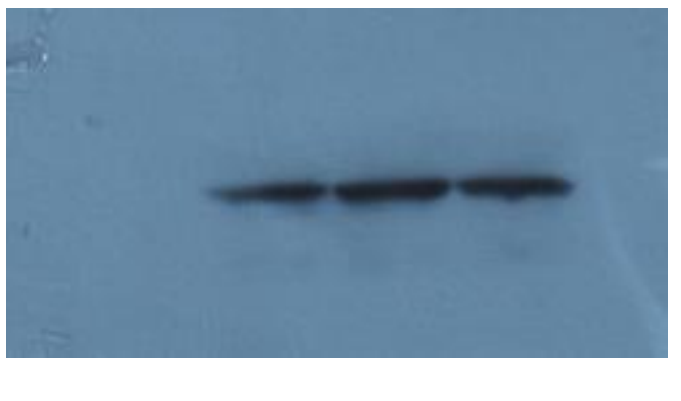

NCOA4

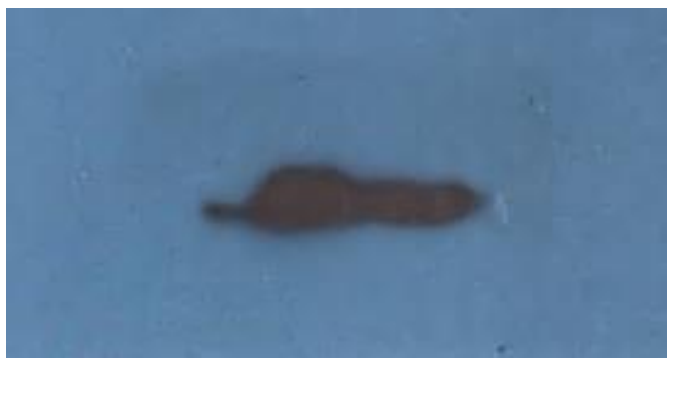

Uncropped blots used in Figure 3 Panel E

SDMe-R

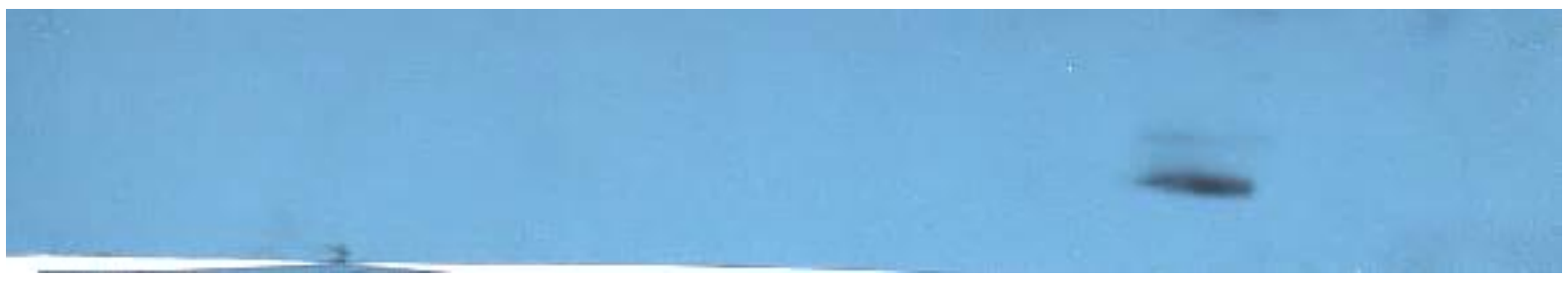

HA

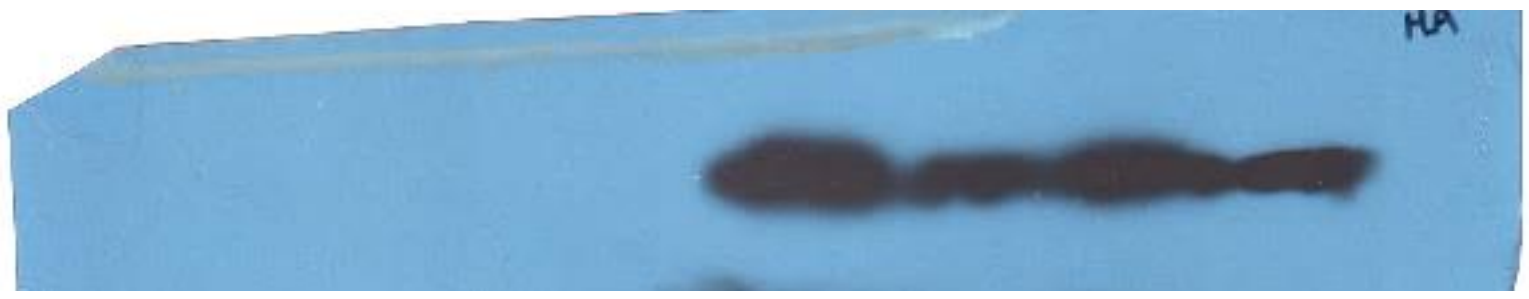

INPUT

ACTB

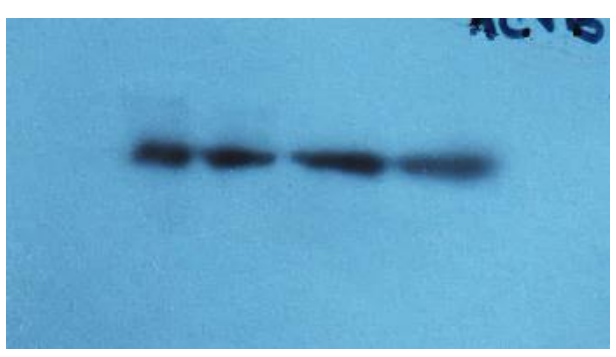

HA

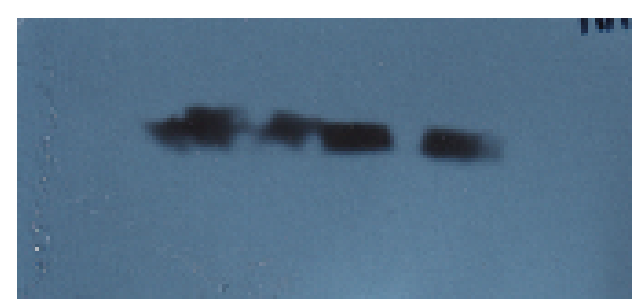

Uncropped blots used in Figure 4 Panel A

FTH1

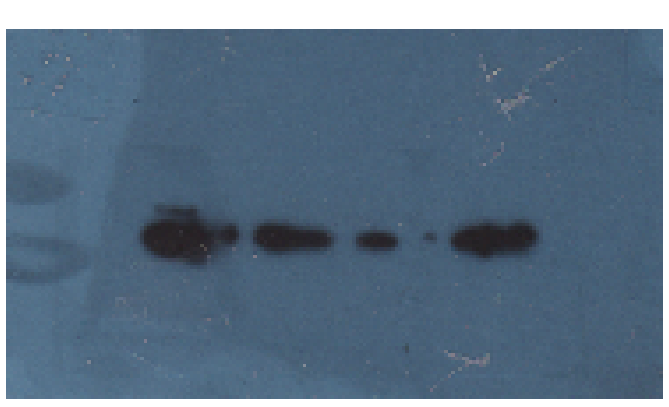

HA

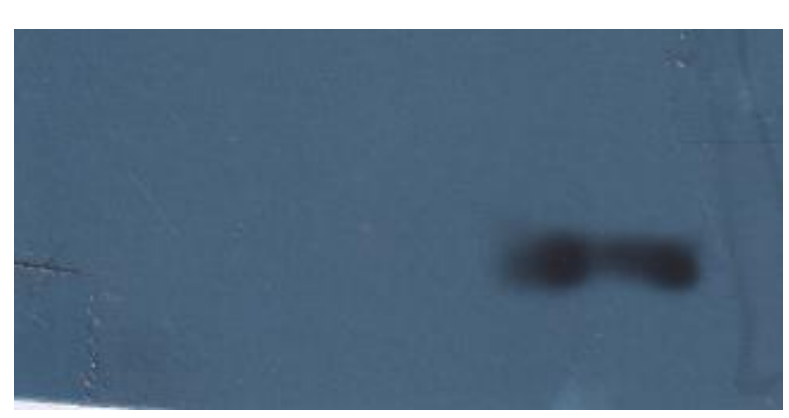

ACTB

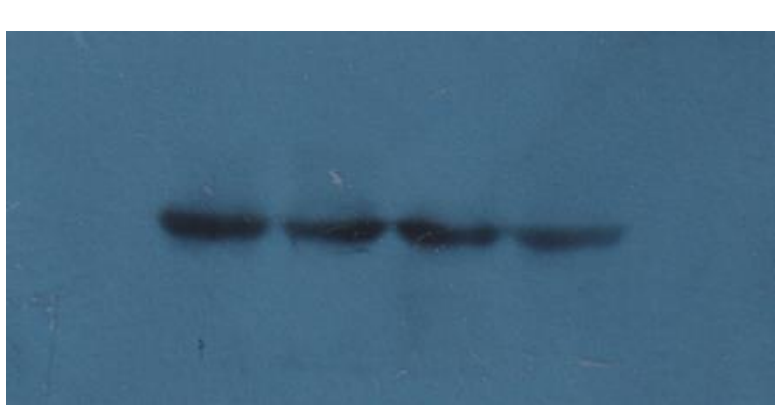

Uncropped blots used in Figure 4 Panel B

NCOA4

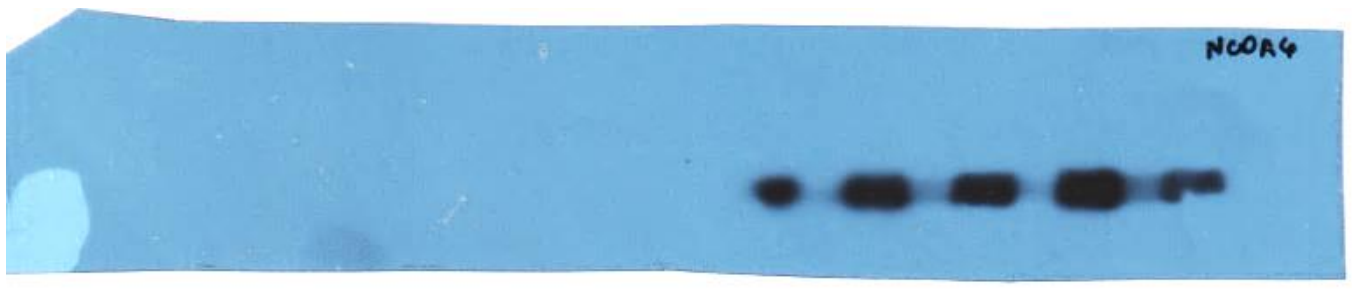

Vitamin D3R

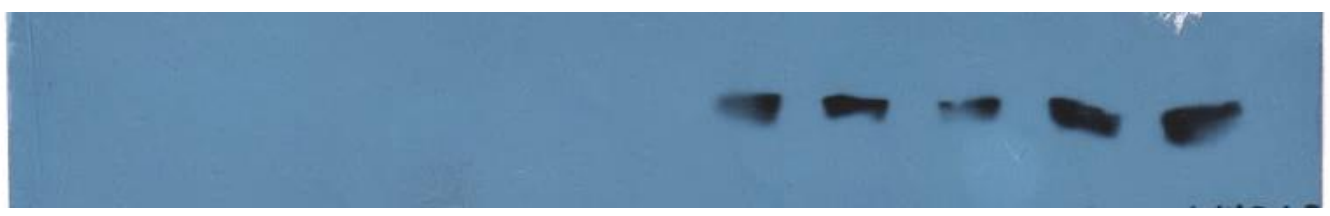

INPUT

ACTB

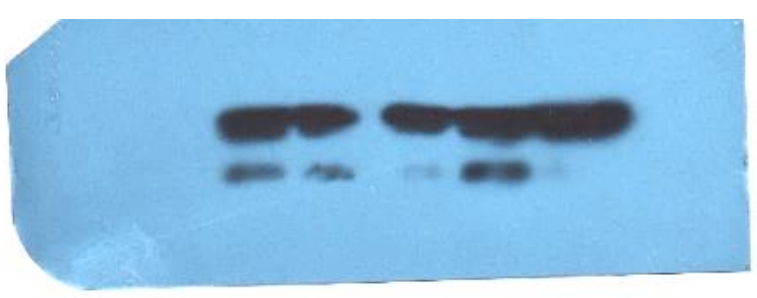

NCOA4

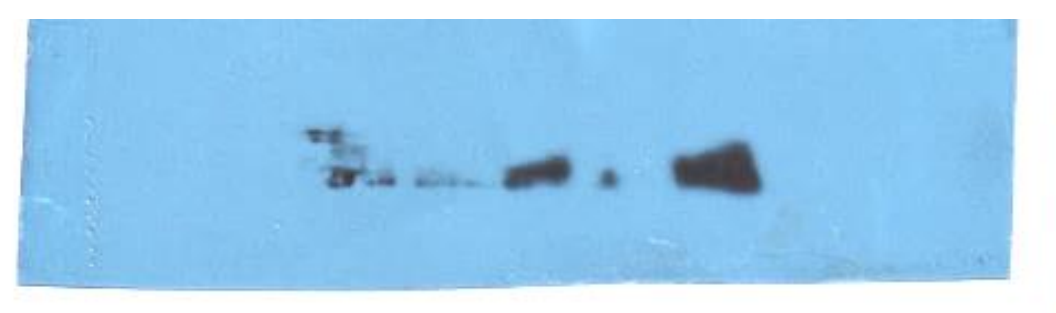

Uncropped blots used in Figure 5 Panel C

GPX4

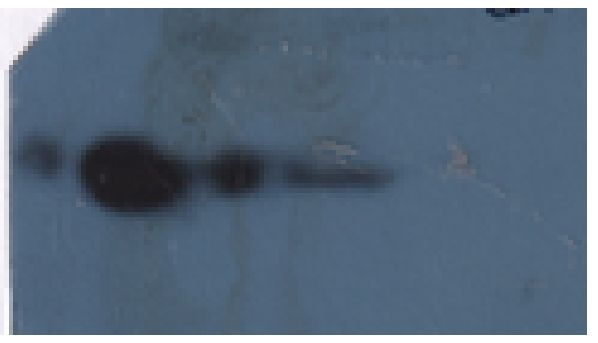

ACTB

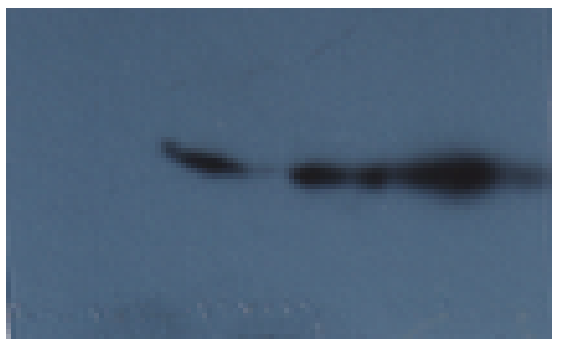

Uncropped blots used in Figure S1 Panel C

FTH1

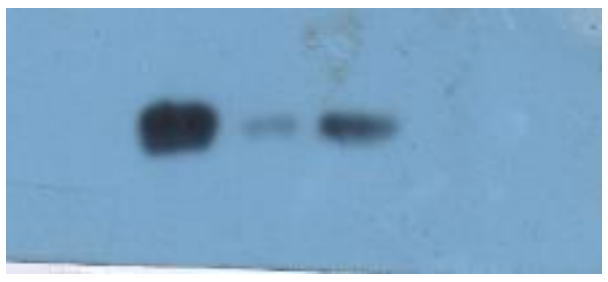

ACTB

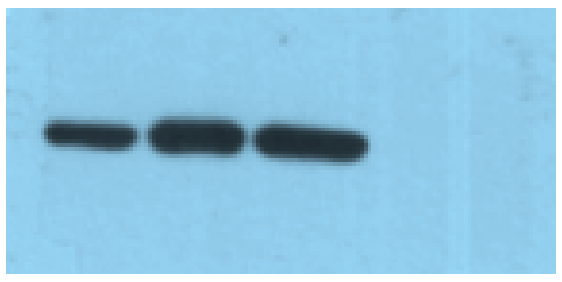

Uncropped blots used in Figure S2 Panel A (left)

FTH1

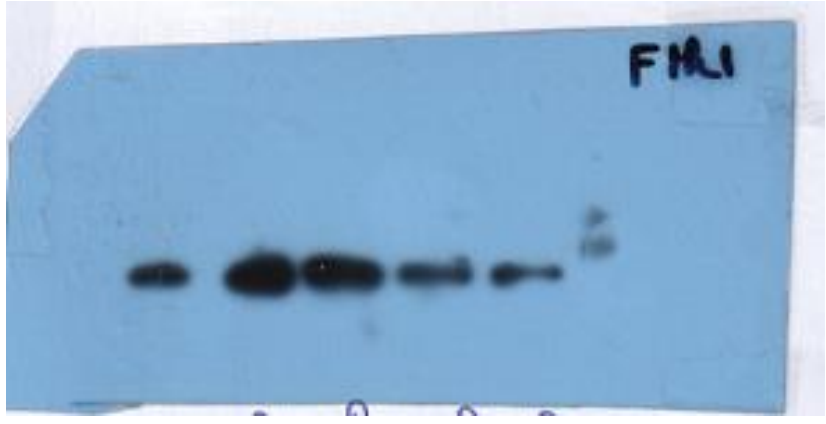

ACTB

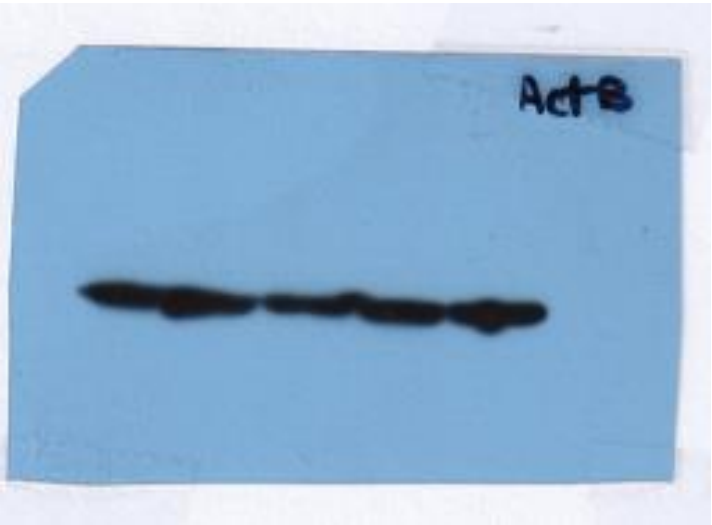

Uncropped blots used in Figure S2 Panel A (right)

FTH1

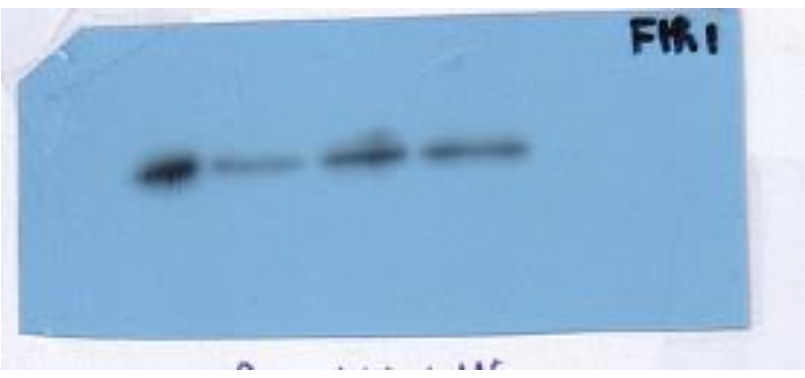

ACTB

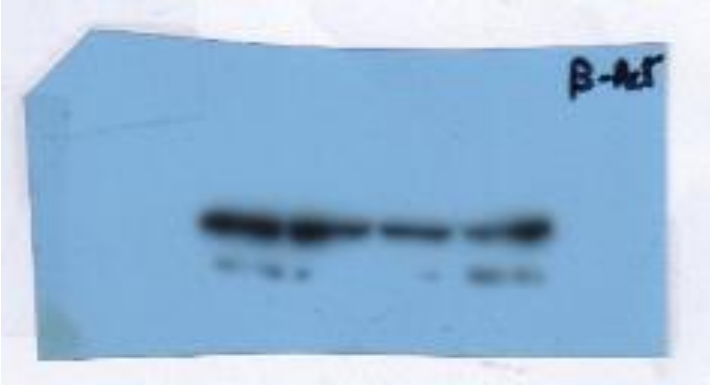

Uncropped blots used in Figure S2 Panel B

NCOA4

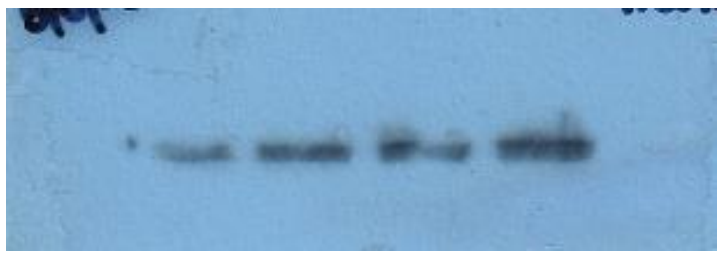

ACTB

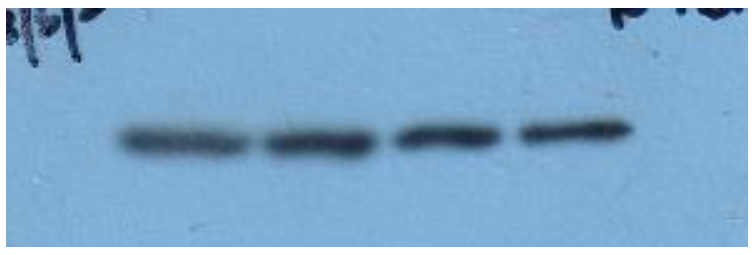

Uncropped blots used in Figure S2 Panel C

FTH1

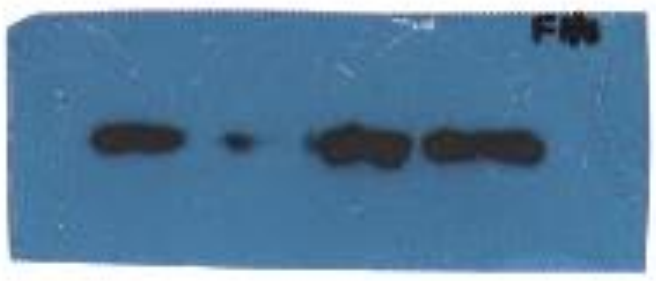

ACTB

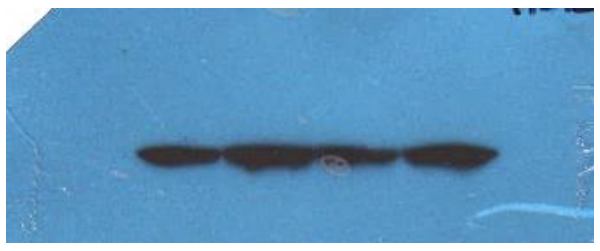

Uncropped blots used in Figure S2 Panel D

FTH1

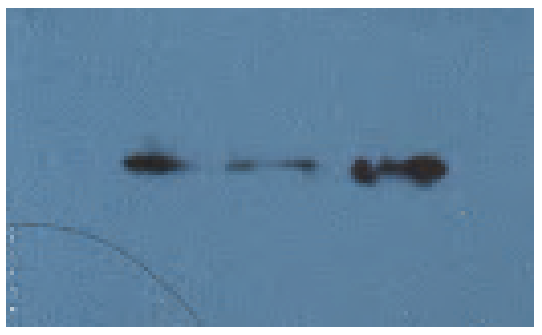

ACTB

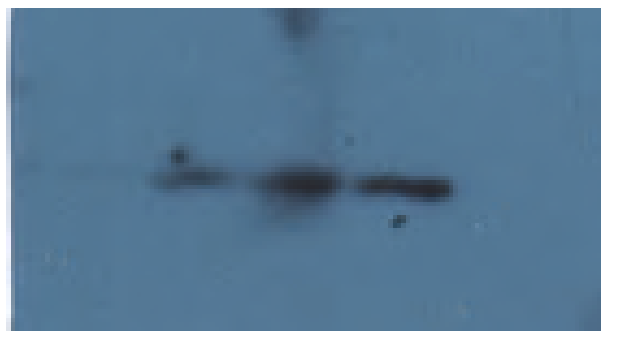

Uncropped blots used in Figure S3 Panel C
